# Visual Screening with Welch Allyn Spot

**DOI:** 10.22336/rjo.2024.23

**Published:** 2024

**Authors:** Camelia Margareta Bogdănici, Irina Andreea Pavel, Cristian Dan Pavel, Alexandru Grigorovici, Coralia-Ada Tătaru

**Affiliations:** * Department of Ophthalmology, “Grigore T. Popa” University of Medicine and Pharmacy, Iași, Romania; ** Department of Histology, Faculty of Medicine, “Grigore T. Popa” University of Medicine and Pharmacy, Iași, Romania; *** Department of Surgery, “Grigore T. Popa” University of Medicine and Pharmacy, Iași, Romania; **** Department of Allergology and Clinical Immunology, “Sf. Spiridon” County Clinical Emergency Hospital, Iași, Romania

**Keywords:** visual screening, autorefractometry, strabismus, refractive errors

## Abstract

**Objective:** Analysis of refractive errors and strabismus deviations following the visual screening of patients with the Welch Allyn Spot device.

**Material and Methods:** This paper is a prospective cross-sectional study of 4281 patients examined with the Welch Allyn Spot device acquired by *Lions Club Romania - District 124*. The study was conducted between May 2019 and August 2021 and was performed with the help of *Lions Club Romania*.

**Results:** In the present study, 4281 patients were evaluated and divided into 5 age groups (6-12 months, 12-36 months, 3-6 years, 6-20 years, and 20-100 years). The most frequent age group was 6-20 years, being identified in 51,97% of participants. We found that the most common refractive error was astigmatism, followed by hyperopia and myopia. Thus, (RE) the refractive errors found in the right eye were: astigmatism 93.23%, hyperopia 4.63%, and myopia 1.05%, and in the left eye (LE): astigmatism 90.40%, hyperopia 6.68%, and myopia 0.84%. Of all participants, 8.81% had horizontal strabismus, esotropia being found in the RE in 4.56% of the participants and the LE in 4.74% of them.

**Conclusions:** The pediatric population was the most affected by astigmatism and esotropia.

**Abbreviations:** RE = right eye, LE = left eye, SD = strabismus deviation

## Introduction

Uncorrected refractive error is the leading cause of visual impairment that produces the second-highest number of years lived with disability [**1**]. Visual deficits from uncorrected refractive errors can have immediate and long-term consequences in children such as poor performance at school and lost employment opportunities. This can further result in an impaired quality of life and low economic gain for individuals, families, and societies [**2**].

Prevalence for myopia, hyperopia, and astigmatism in the global pediatric population is 11.7%, 4.6%, and 14.9%. In the adult population, myopia, hyperopia, and astigmatism are 26.5%, 30.9%, and 40.4%, respectively [**3**]. The estimated pooled prevalence of strabismus, exotropia, and esotropia is 1.93%, 1.23%, and 0.77%, respectively [**4**].

The American Academy of Pediatrics and the National Center for Children’s Vision and Eye Health recommended an instrument-based vision screening for children up to 6 years old as an alternative method when visual acuity cannot be evaluated [**5**,**6**]. The devices provide quick screening results with minimal effort from the examiner or the child [**7**].

This study aims to analyze refractive errors and strabismus deviations following the visual screening of patients with the Welch Allyn Spot device.

## Material and methods

We conducted a prospective, cross-sectional study on 4281 patients divided into five groups according to age:

• 6-12 months; 

• 12-36 months; 

• 3-6 years;

• 6-20 years; 

• 20-100 years. 

The patients were selected between May 2019 and August 2021. The study was performed with the help of *Lions Club Romania – District 124* and the patient screening was carried out with the help of members of *Lions Clubs* in Iași (*Lions D Club Iași* and *Lions Iași* 2000), Constanța, Timișoara and Bacău.

The main device used for this screening was the Welch Allyn Spot. Data collected from each patient included: age group, refractive values of both eyes (sphere and cylinder), and strabismus deviations (esotropia and exotropia). Following screening, additional investigations and treatment were carried out for patients with eye conditions.

After enrolling patients, all parameters used in the study were recorded in a standardized database (Microsoft Excel 2013) and appropriately coded. The data were systematized and centralized in an SPSS 18.0 database and were processed with the statistical functions for which they were suitable.

## Results

**Fig. 1:**
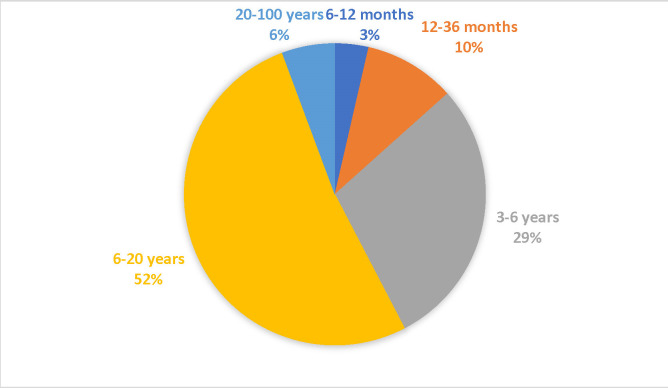
Structure of groups depending on age

In our study, 4281 patients were evaluated and were divided into 5 age groups (6-12 months, 12-36 months, 3-6 years, 6-20 years, and 20-100 years). The age category with the highest percentage was the 6-20 years group with 51.97%, followed by the 3-6 years group with 28.97% (**Fig. 1**). 

The most frequent refractive error found in our study was astigmatism, being diagnosed in more than 90% of both eyes, followed by hyperopia and myopia with less significant values. In the analysis, the right eye (93.23%) of the participants was more affected by astigmatism than the left eye (90.40%) having a difference of almost 3%. Hyperopia was assessed in the right eye in 4.63%, in the left eye in 6.68%, and myopia in 1.05% and 0.85%, respectively. Patients with emmetropia had the following percentages on their right and left eye: 1.10% and 2.08% (**Table 1**).

**Table 1:** Distribution of cases with refractive errors and emmetropia by eye

Refractive error/ Emmetropia	RE		LE	
	Number of cases	%	Number of cases	%
Hyperopia	198	4.63%	286	6.68%
Myopia	45	1.05%	36	0.84%
Astigmatism	3991	93.23%	3870	90.40%
Emmetropia	47	1.10%	89	2.08%
Total	4281	100%	4281	100%

An important aspect of refractive errors is their dioptric values. In the case of stigmatic refractive errors, the most prevalent subtypes were low hyperopia with a percentage for the RE of 4.53% and the LE of 6.63%, and low myopia with a percentage below 1% for both eyes. The other subtypes were found in a very small number, with insignificant percentages (**Table 2**).

**Table 2:** Distribution of astigmatism refractive errors by value in each eye

Stigmatic refractive error subtypes	Reference range value	LE		RE	
		Number of cases	%	Number of cases	%
Low hyperopia	0 - (+3) D	194	4.53%	284	6.63%
Moderate hyperopia	(+3) - (+6)	3	0.07%	0	0.00%
High hyperopia	> (+6) D	1	0.02%	2	0.05%
Low myopia	(-3) - 0 D	41	0.96%	34	0.79%
Moderate myopia	(-3) - (-6) D	1	0.02%	0	0.00%
High myopia	< (-6)	3	0.07%	2	0.05%
Total	-	4281	100%	4281	100%

As for astigmatism, the classification was made according to both spherical and cylindrical power. In the first case, the most prevalent types of astigmatism were low and moderate mixed astigmatism, low compound myopic astigmatism, and simple myopic astigmatism with the percentages for the right eye of 85.90%, 3.70%, 8%, and 7.24%, and for the left eye 85.74%, 3.72%, 8.14% and 7.60%, respectively (**Table 3**). In the second case, the cylinder power, whose range was (-0.75) - 0 D, also called low astigmatism, was the most prevalent and was found in 65% of both eyes. The other two, moderate and high astigmatism, with ranges (-1) - (-2.5) D and > (-2.5) D, are found in a smaller proportion. Moderate and high astigmatism was estimated to be 23.15% and 5.61% for the right eye and 20.25% and 4.67% for the left eye (**Table 4**). The approximately equal results of the two eyes mark the dioptric balance.

**Table 3:** Astigmatism types according to the spherical value

Astigmatism types	Reference range value	LE		RE	
		Number of cases	%	Number of cases	%
Low mixt astigmatism	0 - (+3) D	3180	85.90%	3066	85.74%
Moderate mixt astigmatism	(+3) - (+6)	137	3.70%	133	3.72%
High mixt astigmatism	> (+6) D	17	0.46%	19	0.53%
Low compound myopic astigmatism	(-3) - 0 D	296	8.00%	291	8.14%
Moderate compound myopic astigmatism	(-3) - (-6) D	51	1.38%	49	1.37%
High compound myopic astigmatism	< (-6)	21	0.57%	18	0.50%
Simple miopic astigmatism	0 D	289	7.24%	294	7.60%
Total		3991	100.00%	3870	100.00%

**Table 4:** Astigmatism types according to the cylindrical value

Astigmatism types	Reference range value	LE		RE	
		Number of cases	%	Number of cases	%
Low astigmatism	(-0.75) - 0 D	2760	64.47%	2803	65.48%
Moderate astigmatism	(-1) - (-2.5) D	991	23.15%	867	20.25%
High astigmatism	> (-2.5) D	240	5.61%	200	4.67%
Total	-	4281	100%	4281	100%

In the current paper, we calculated the mean, standard deviation, and spherical equivalent for astigmatism for each eye. In the RE, the results were as follows: for hyperopia: 0.60 ± 0.78 D, myopia: -1.23 ± 1.82 D and astigmatism: -0.84 ± 0.79 D, and in the LE for hyperopia: 0.48 ± 0.64 D, myopia: -0.89 ± 1.58 D and astigmatism: -0.79 ± 0.77 D.

Due to the increased number of patients with astigmatism, we considered necessary a much broader statistical analysis. We calculated the percentage of patients who had only one eye affected by astigmatism and the one in which both were affected by astigmatism. Patients with only one eye affected were 98.23%, and those with both eyes affected were 85.31%. 

In addition, we evaluated the following types of astigmatism: simple myopic astigmatism, compound myopic astigmatism, and mixed astigmatism. Mixed astigmatism was the most prevalent type of astigmatism with 85% for both eyes. The compound myopic and simple myopic were associated with approximate percentages of 9% and 7%, respectively.

Overestimated astigmatism occurred when the astigmatism values measured with the Welch Allyn Spot device were higher than the patient’s actual values. To demonstrate this, we calculated the number of patients whose astigmatism was characterized by spherical power equal to 0 and cylindrical power different from 0 (**Table 5**).

**Table 5:** Distribution of overestimated astigmatism cases

	RE	LE
Number of cases	289	294
%	6.75%	6.87%

Anisometropia had a prevalence of 7.83% and was found in most cases in patients with astigmatism (7.26%) (**Table 6**). Analyzing the cases of anisometropia according to age group, we found that the 6-20 years age group had the highest percentage (50.75%), followed by the 3-6 years group (20.30%).

**Table 6:** Distribution of anisometropia cases

	Anisometropia				
	Astigmatism both eyes	Other refractive errors	Total anisometropia cases	No anisometropia	Total
Number of cases	311	24	335	3946	4281
%	7.26%	0.56%	7.83%	92.17%	100%

In this study, the percentage of individuals who were affected by strabismus was 8.81%, of which 2.32% had strabismus in both eyes, and 6.49% represented the percentage of patients with strabismus deviation in only one eye. 

The most affected age groups in the pediatric population affected by strabismus were: 6-20 years (37.14%), 3-6 years (29.71%), and 12-36 months (26.26%). 

The most common type of strabismus deviation was esodeviation found in 4.56% of the RE and 4.74% of the LE. Exodeviation was found in 1.05% and 0.77% (**Table 7**). In addition, we examined the relationship between strabismus and astigmatism. It has been shown that astigmatism was associated more often with esodeviation than with exodeviation (**Table 8**).

**Table 7:** Distribution of strabismus cases

Strabismus deviation (SD)	RE		LE	
	Number of cases	%	Number of cases	%
Esodeviation	195	4.56%	203	4.74%
Exodeviation	45	1.05%	33	0.77%
No SD	4041	94.39%	4045	94.49%
Total	4281	100.00%	4281	100.00%

**Table 8:** Associations between strabismus and astigmatism

Strabismus deviation (SD)	Astigmatism RE		Astigmatism LE	
	Number of cases	%	Number of cases	%
Esodeviation	192	4.48%	200	4.67%
Exodeviation	43	1.00%	33	0.77%
Total	4281	100.00%	4281	100.00%

## Discussions

The main device used for this screening was the Welch Allyn Spot ophthalmic device. This screening device is used in ophthalmological practice and provides a simultaneous binocular assessment of refraction, pupil size, interpupillary distance, and eye deviation. The device is used on patients from 6 months of age [**8**].

In a 2018 study, 134 patients aged 7 to 50 years were evaluated with both Spot Vision Screening and clinical refractometry under cycloplegia. The results showed that the difference between the two assessment methods, expressed in spherical equivalents, was +0.66 ± 0.56 D, and supported the use of Spot Vision Screening as an associated alternative method for refraction assessment [**9**].

In a 2017 study by Forcina et al., a group of 184 children younger than 3 years was evaluated. They were examined with the Welch Allyn Spot device and then underwent an ophthalmic examination including cycloplegic refraction and sensory testing within 6 months of the date of testing with the device by a pediatric ophthalmologist. Compared to the ophthalmologist’s examination, the device successfully detected 89.7% of patients, with an overall sensitivity of 89.8% and a specificity of 70.4%. According to the study, the Welch Allyn Spot achieved good sensitivity and specificity for detecting amblyogenic risk factors in this young cohort, providing real support for automated vision screening in young children [**10**].

Another study, this time of children over 6 years old, showed that the device achieved 89.5% sensitivity and 76.7% specificity. The study was conducted on 330 children, of whom, the Welch Allyn Spot device detected 313 (95%). In conclusion, it was found that screening with this device in this age group is an acceptable refractive method for detecting significant refractive errors [**7**]. In our research, the most frequent refractive error found was astigmatism, being diagnosed more than 90% in both eyes, followed by hyperopia and myopia with less significant values.

The Welch Allyn Spot device overestimates astigmatism and myopia and underestimates hyperopia [**11**,**12**]. We analyzed overestimating astigmatism by associating the cylindrical power less than 0 D with the spherical power equal to 0 D. The most important causes of this phenomenon are keratoconus, cataract, small pupil, pseudophakia, high hyperopia, pterygium involving the visual axis, microcornea, adherent leukoma and corneal scar [**12**]. In the current study, we found that astigmatism was overestimated in about 7% of cases. 

A study by Peterseim focused on strabismus detection with the Spot Vision Screener. A total of 444 children were included (mean age 72 months). Of them, 20.9% were found to have strabismus on examination by the pediatric ophthalmologist. The sensitivity of the device for detecting strabismus was 77.17% and the specificity was 93.73%. Thus, the study demonstrates good sensitivity and excellent specificity of the device for strabismus detection [**13**]. In this study, 8.81% of the participants were affected by strabismus (2.32% had strabismus in both eyes and 6.49% in one eye). The most common type of strabismus deviation was esodeviation found in 4.56% of the RE and 4.74% of the LE.

In our research, anisometropia had a prevalence of 7.83% and was found in most cases in patients with astigmatism (7.26%). A study conducted by Huynh et al. on 1765 children, mostly 6-year-olds, showed a prevalence of anisometropia of 1.6% [**14**]. Therefore, the values in our study were higher. 

The same study also showed that strabismus of all types (exotropia and esotropia) and amblyopia were closely related to the presence of anisometropia [**14**]. Although amblyopia was not investigated in our study, it is an important research topic because patients with amblyopia may have difficulties related to socio-professional integration [**15**,**16**].

The prevalence of anisometropia increases with the educational stage, with the highest rate being found in adolescents [**17**]. Analyzing the cases of anisometropia in our study according to age group, we found that the 6-20 age group had the most significant percentage of 50.75%, followed by the 3-6 age group with 20.30%. These age groups are most likely to develop strabismus and amblyopia because they belong to the critical age at which vision develops. A study conducted by Deng and Gwiazda, which investigated anisometropia in children aged 6 months to 15 years, found that the prevalence of anisometropia was 1.96%, 1.27%, and 5.77% at 6 months, 5 years, and 12 to 15 years, respectively [**18**]. Thus, the age group with the highest prevalence was 12 to 15 years.

A study conducted in 2021, in which 22887 participants older than 5 years were included, concluded that astigmatism had a significant association with exodeviation [**19**]. Another study found that clinically significant exodeviation was associated with amblyopia, a family history of strabismus, and astigmatism [**20**]. Compared to these studies, in our research, we found that esodeviation was most closely related to astigmatism, in RE 4.48% and LE 4.67%.

The strength of this study was that vision screening is a simple and innovative method for detecting pathologies such as refractive errors and strabismus. The Welch Allyn device helps specialists to detect them and it can be carried out by medical staff.

One of the study’s limitations was that we did not assess visual acuity to diagnose amblyopia and screening could not be performed on children under 6 months of age.

## Conclusions

The Welch Allyn Spot screening device is a real help for ophthalmologists in detecting strabismus and refractive errors in patients over 6 months of age. Thanks to this technology, specialists obtain early diagnoses and improve the quality of medical acts in ophthalmology.

The ophthalmological screening was carried out with the support of *Lions Club Romania* members, which led to the detection of important pathologies in children. The pediatric population was the most affected by both strabismus and refractive errors. If untreated, these two pathologies harm pediatric patients, favoring the development of amblyopia and causing decreased visual acuity. 

**Conflict of Interest Statement**

The authors state no conflict of interest.

**Informed Consent and Human and Animal Rights Statement**

Informed consent has been obtained from all individuals and legal guardians of the individuals included in this study.

**Authorization for the use of human subjects**

Ethical approval: The research related to human use complies with all the relevant national regulations, and institutional policies, as per the tenets of the Helsinki Declaration, and has been approved by the Ethics Committee of “Grigore T. Popa” University of Medicine and Pharmacy, Iași, Romania (139/17.08.2015).

**Acknowledgments**

None.

**Sources of Funding**

None.

**Disclosures**

None.
